# Inhibition of c-FLIP_L_ expression by miRNA-708 increases the sensitivity of renal cancer cells to anti-cancer drugs

**DOI:** 10.18632/oncotarget.7149

**Published:** 2016-02-03

**Authors:** Eun-Ae Kim, Sang-Woo Kim, Jehyun Nam, Eon-Gi Sung, In-Hwan Song, Joo-Young Kim, Taeg Kyu Kwon, Tae-Jin Lee

**Affiliations:** ^1^ Department of Anatomy, College of Medicine, Yeungnam University, Nam-gu, Daegu, Republic of Korea; ^2^ Department of Biological Sciences, Pusan National University, Busan, Republic of Korea; ^3^ Department of Immunology, School of Medicine, Keimyung University, Dalseo-Gu, Daegu, Republic of Korea

**Keywords:** c-FLIP, miR-708, RCC, doxorubicin

## Abstract

Dysregulation of the anti-apoptotic protein, cellular FLICE-like inhibitory protein (c-FLIP), has been associated with tumorigenesis and chemoresistance in various human cancers. Therefore, c-FLIP is an excellent target for therapeutic intervention. MicroRNAs (miRNAs) are small non-coding RNAs that are involved in tumorigenesis, tumor suppression, and resistance or sensitivity to anti-cancer drugs. However, whether miRNAs can suppress c-FLIP_L_ expression in cancer cells is unclear. The aim of this study was to identify miRNAs that could inhibit the growth of renal cancer cells and induce cell death by inhibiting c-FLIP_L_ expression. We found that MiRNA-708 and c-FLIP_L_ expression were inversely correlated. While c-FLIP_L_ expression was upregulated, miRNA-708 was rarely expressed in renal cancer cells. Luciferase reporter assays demonstrated that miRNA-708 negatively regulated c-FLIP_L_ expression by binding to the miRNA-708 binding site in the 3′ untranslated region (3′UTR) of c-FLIP_L_. Ectopic expression of miRNA-708 increased the accumulation of sub-G1 populations and cleavage of procaspase-3 and PARP, which could be prevented by pretreatment with the pan-caspase inhibitor, Z-VAD. Ectopic expression of miRNA-708 also increased the sensitivity to various apoptotic stimuli such as tumor necrosis factor-related apoptosis-inducing ligand, doxorubicin (Dox), and thapsigargin in Caki cells. Interestingly, miRNA-708 specifically repressed c-FLIP_L_ without any change in c-FLIP_s_ expression. In contrast, inhibition of endogenous miRNA-708 using antago-miRNAs resulted in an increase in c-FLIP_L_ protein expression. The expression of c-FLIP_L_ was upregulated in renal cell carcinoma (RCC) tissues compared to normal tissues. In contrast, miRNA-708 expression was reduced in RCC tissues. Finally, miRNA-708 enhanced the tumor-suppressive effect of Dox in a xenograft model of human RCC. In conclusion, miRNA-708 acts as a tumor suppressor because it negatively regulates the anti-apoptotic protein c-FLIP_L_ and regulates the sensitivity of renal cancer cells to various apoptotic stimuli.

## INTRODUCTION

MicroRNAs (miRNA) are small non-protein-coding RNAs of 19–25 nucleotides that negatively regulate the expression of multiple target genes by inducing mRNA degradation or translational repression [1, 2]. Recent data has shown that miRNAs play important roles in various biological processes such as cellular differentiation, proliferation, and death [3]. MiRNAs are expressed aberrantly in many human cancers and play significant roles in tumor development, tumor suppression, metastasis, and resistance or sensitivity to anti-cancer drugs [4]. MiRNAs can act as oncogenes or tumor suppressor genes; they can function as oncogenes by repressing tumor suppressors, and they can function as tumor suppressors by negatively regulating oncogenes [3, 5]. Therefore, studies of tumor suppressive miRNAs and their respective target genes are important in order to understand miRNA-regulated cancer pathways.

Cellular FLICE-like inhibitory protein (c-FLIP), a catalytically inactive caspase-8/-10 homologue, inhibits caspase-8 recruitment and processing at the death-inducing signaling complex (DISC) [6]. Overexpression of c-FLIP has been shown to protect against apoptosis mediated by death receptors including FasL and tumor necrosis factor-related apoptosis-inducing ligand (TRAIL), and mediated by the mitochondria in a wide range of human cancers [7–11]. Overexpression of c-FLIP has been observed in a wide variety of human cancers and can confer resistance to chemotherapeutic agents, alter cell cycle progression, and enhance cell proliferation and carcinogenesis [10, 12–16]. These data suggest that c-FLIP is a promising therapeutic target. Two splice variants are encoded by c-FLIP: the long form (c-FLIP_L_) and the short form (c-FLIP_s_) [17]. These c-FLIP variants differentially regulate apoptosis [18]. The levels of c-FLIP are regulated at both the transcriptional and post-translational levels. In addition, c-FLIP is regulated by post-transcriptional events. For example, miRNA-512-3p has been shown to regulate c-FLIP expression in hepatocellular carcinoma cells [19]. The c-FLIP expression was also inhibited by mi-RNAs hsa-miR-1246, hsa-miR-320a and hsa-miR-196b-5p were induced in human umbilical vein endothelial cells [20]. Recently, it was demonstrated that c-FLIP_L_ and c-FLIP_s_ have different functions in cell death pathways such as apoptosis, necroptosis, and autophagy [21–25].

The aim of this study was to identify novel miRNAs that regulate c-FLIP expression and are involved in renal carcinogenesis. We identified miRNA-708 (miR-708), a tumor suppressive miRNA that targets c-FLIP_L_, and investigated the effects of this miRNA on the anti-cancer drug-induced cell death of human renal cancer cells.

## RESULTS

### c-FLIP is a functional target of miR-708 in RCC

**Figure 1:**
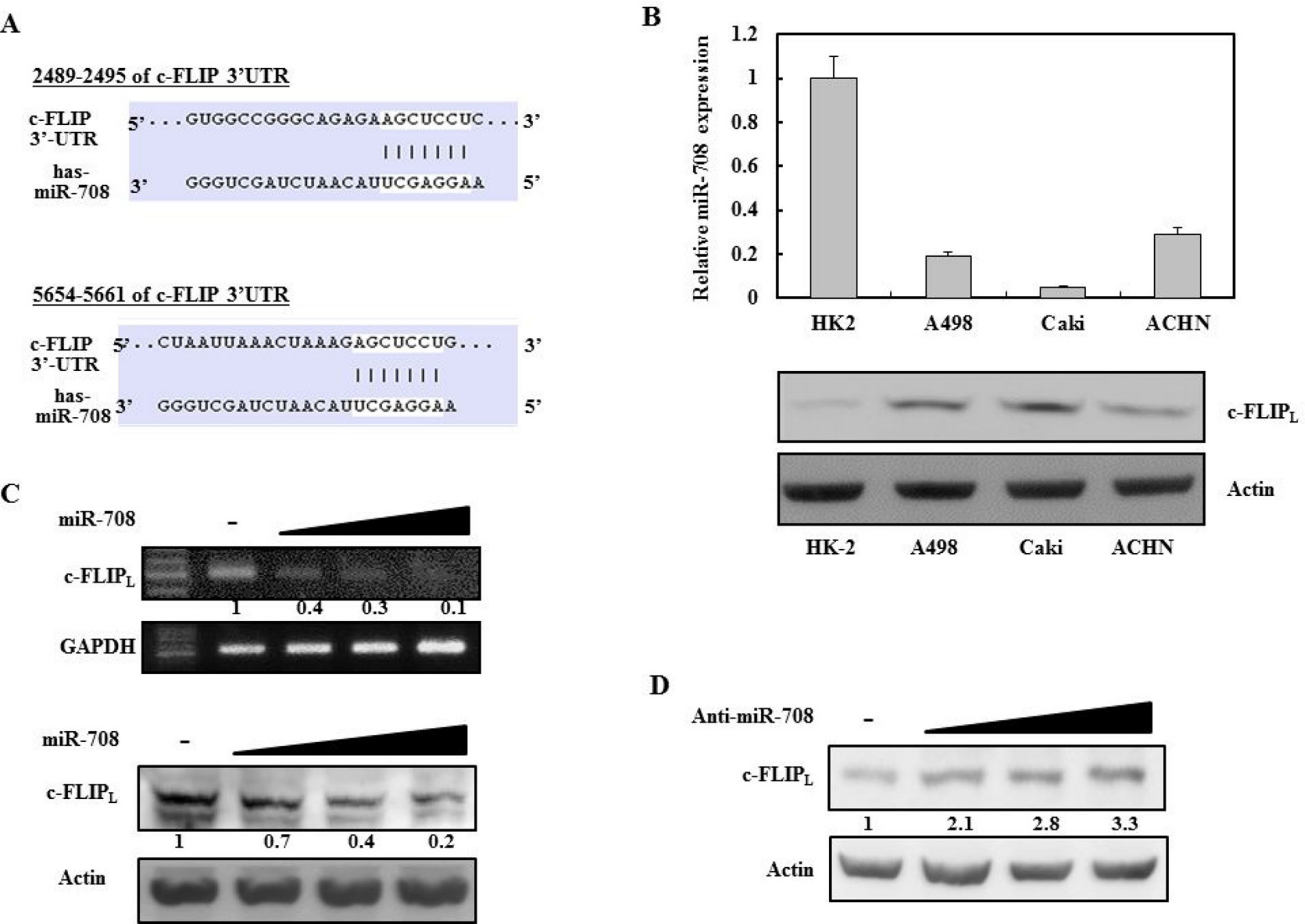
Identification of novel miRNAs that regulate c-FLIP_L_ expression in renal cancer cells **A.** MiR-708 binding sites in the 3′UTR of c-FLIP_L_ mRNA. **B.** Quantitative RT-PCR analysis of relative miR-708 expression levels in human renal cancer cell lines (A498, Caki, and ACHN) and a normal immortalized renal cell line (HK2) (Upper panel). Relative c-FLIP_L_ expression in human renal cancer cell lines and a normal renal cell line (Bottom panel). **C.** RT-PCR analysis of c-FLIP_L_ mRNA in Caki cells transfected as indicated (Upper panel). Immunoblots for c-FLIP_L_ in Caki cells transfected as indicated. Actin was used as the loading control (Bottom panel). **D.** Immunoblots for c-FLIP_L_ in Caki cells transfected as indicated.

A bioinformatic analysis program, TargetScan, was used to identify miRNAs that specifically suppress c-FLIP_L_ expression. The TargetScan miRNA target predictions showed that the c-FLIP_L_ 3′ 2-UTR contained two potential binding sites for miR-708 at nucleotides 2489 and 5654 (Fig. 1A, http://www.targetscan.org/cgi-bin/targetscan/vert_61/view_gene.cgi?taxid=9606&rs=NM_001127183&members=&showcnc=0&shownc=0&showncf=). Analysis of miR-708 expression in renal cell lines revealed that miR-708 expression was suppressed in renal cancer cell lines, whereas c-FLIP_L_ was upregulated in renal cancer cells compared to normal renal (HK2) cells (Fig. 1B). To determine whether exogenous miR-708 could repress c-FLIP_L_ expression, Caki cells, which have high c-FLIP_L_ levels, were analyzed. After the cells were transiently transfected with the indicated concentrations of either premature miR-708 for 24 hours or a control miRNA (miR-cont), the expression of c-FLIP_L_ was analyzed by reverse transcriptase polymerase chain reaction (RT-PCR) and Western blotting (Fig. 1C). As shown in Fig. 1C, ectopic expression of miR-708 inhibited c-FLIP_L_ mRNA and protein expression in a dose-dependent manner. In contrast, transfection with anti-miR-708 resulted in an increase in c-FLIP_L_ expression in Caki cells (Fig. 1D).

**Figure 2:**
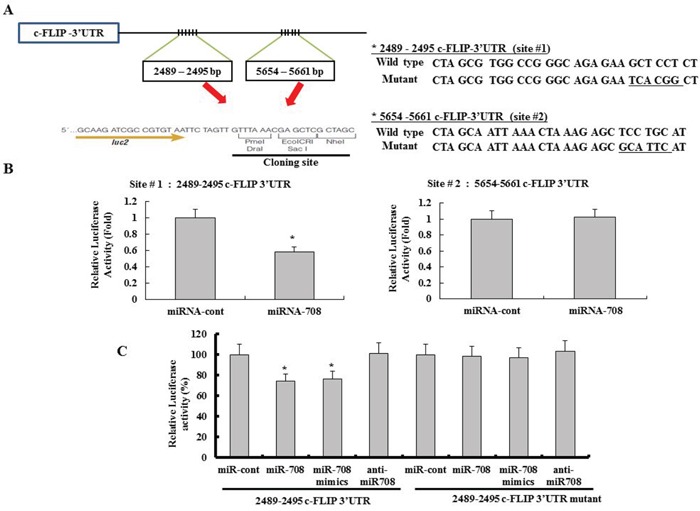
The c-FLIP_L_ isoform is a functional target of miR-708 in renal cancer **A.** Schematic representation of c-FLIP_L_ showing putative miR-708 target sites (Left panel). Also shown are the nucleotides mutated in c-FLIP_L_ 3′UTR mutant (Right panel). **B.** Luciferase activity assay with the respective wild-type luciferase constructs containing two different miR-708 target sequences (site #1 or site #2) transfected with miR-cont or miR-708. For all luciferase activity assays, firefly luciferase values were normalized to renilla luciferase activity and plotted as the relative luciferase activity. The data is reported as the mean ± SD (n = 3). Statistics, Student's t-test for unpaired values. * indicates P < 0.05 versus miR-cont-transfected cells. **C.** Luciferase assays showing repression of the wild-type UTR (c-FLIP_L_ 3′-UTR) or mutant UTR (c-FLIP_L_ 3′UTR-mutant) following transfection of miR-708, miR-708 mimics, anti-miR-708, and miR-cont. The data is reported as the mean ± SD (n = 3). * indicates P < 0.05 versus miR-cont-transfected cells.

We next investigated whether the 3′-UTR of c-FLIP_L_ was a functional target of miR-708 in RCC. Because miR-708 could bind to two different regions of the 3′-UTR of c-FLIP_L_ mRNA (Fig. 1A), we investigated which of the two regions was involved in miR-708 binding. The predicted miRNA-binding sequences of c-FLIP_L_ (sites 1 and 2) were cloned into the downstream region of a luciferase reporter construct (pmirGLO-c-FLIP #1 and pmirGLO-c-FLIP #2, Fig. 2A). Caki cells were transfected transiently with these constructs in the presence of either pre-miR-708 or miR-cont. As shown in Fig. 2B, miR-708 significantly reduced the luciferase activities of pmirGLO-c-FLIP #1 compared to miR-cont, but miR-708 failed to affect the luciferase activity of pmirGLO-c-FLIP #2. These data suggested that miR-708 specifically bound to the 3-UTR of c-FLIP_L_ at nucleotide 2489 and impaired c-FLIP_L_ expression. In addition, miR-708 mimics significantly reduced the luciferase activity compared to miR-cont. In contrast, the luciferase activity of the reporter vector containing a mutated 3′-UTR in c-FLIP_L_ was unaffected by miR-708 (Fig. 2C).

### MiR-708 inhibits renal cancer cell proliferation and promotes apoptosis

**Figure 3:**
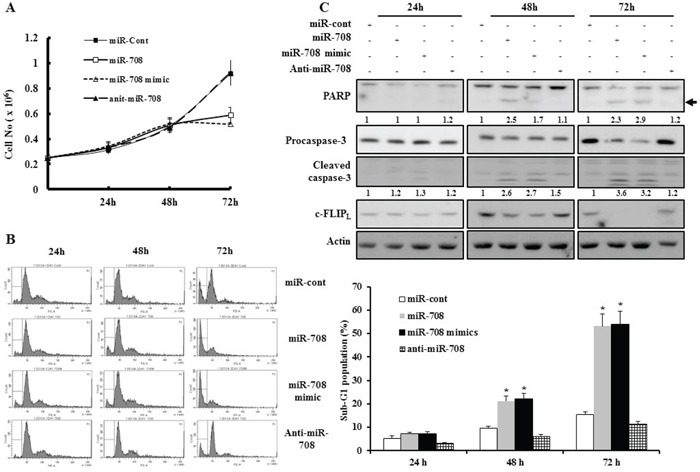
MiR-708 inhibits renal cancer cell proliferation and promotes apoptosis **A.** Caki cell proliferation analyzed using trypan blue dye exclusion assays 24 h, 48 h, and 72 h after transfection with miR-708, miR-708 mimics, anti-miR-708, or miR-cont. Cell proliferation was significantly inhibited after transfection with miR-708 or miR-708 mimics compared to cells transfected with miR-cont. **B.** Caki cells were transfected with miR-708, miR-708 mimics, anti-miR-708, or miR-cont for 24 h, 48 h, or 72 h. Apoptosis was analyzed as a sub-G1 fraction by FACS (Histogram, left panel). Flow cytometry analysis of apoptotic cells (Graph, right panel). The data is reported as the mean ± SD (n = 3). * indicates P < 0.05 versus miR-cont-transfected cells. **C.** Transfection with miR-708 or miR-708 mimics induced cleavage of PARP and procaspase-3 after 48 h. Equal amounts of cell lysates (40 μg) were subjected to electrophoresis and analyzed by Western blotting with c-FLIP_L_, PARP, and caspase-3 antibodies. Proteolytic cleavage of PARP is indicated by the arrow. Actin was used as a loading control in all immunoblots. The relative levels of cleaved PARP and caspase-3 in miR-transfected cells were expressed as the ratio of the densitometric values for each protein to those of actin.

To examine the functional significance of miR-708 in RCC, renal cancer cells were transfected with miR-708. Cell proliferation was significantly inhibited in Caki cells after transfection with miR-708 compared to miR-cont-transfected cells (Fig. 3A). The percentage of the sub-G1 population markedly increased in response to miR-708 transfection compared to miR-cont transfection of Caki cells 48 hours after transfection (Fig. 3B). We next examined whether transfection with miR-708 resulted in the activation of caspases in Caki cells. Forced expression of miR-708 in Caki cells led to a significant decrease in the protein levels of caspase-3 precursors, an increase in cleaved caspase-3 levels, and concomitant cleavage of PARP 48 h after transfection (Fig. 3C). Similarly, transfection with the miR-708 mimics resulted in a significant increase in the accumulation of the sub-G1 phase and activation of caspase pathways compared to miR-cont-transfected cells (Fig. 3C). Ectopic expression of miR-708 also decreased the clonogenicity of Caki cells compared to miR-cont-expressing cells (Supplementary Fig. 1).

### miR-708-mediated downregulation of c-FLIP_L_ enhances sensitivity to various apoptotic stimuli

**Figure 4:**
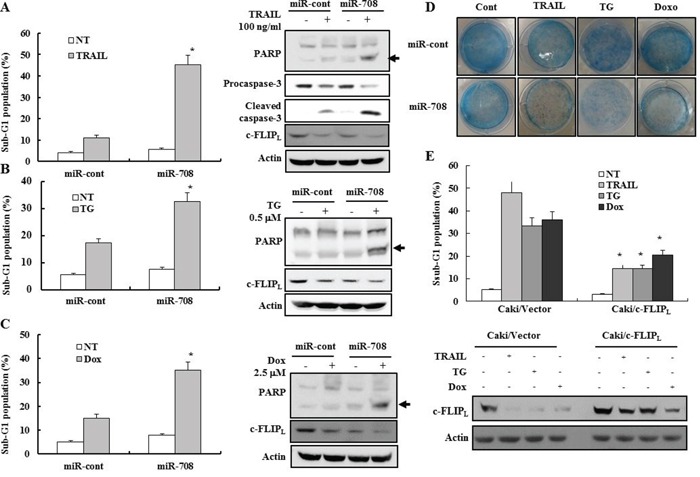
MiR-708-mediated downregulation of c-FLIP_L_ enhances sensitivity to various apoptotic stimuli **A-C.** Caki cells transfected with miR-cont or miR-708 were treated with TRAIL, TG, or Dox for 24 h. The cellular DNA content was then measured after propidium iodide staining. (A) Apoptosis was analyzed as a sub-G1 fraction by FACS. The data is reported as the mean ± SD. (n = 3). * indicates P < 0.05 versus the miR-cont-transfected cells (Left panel). Cells treated as described above were harvested in a lysis buffer and equal amounts of cell lysates (40 μg) were subjected to electrophoresis and analyzed by Western blotting with PARP, procaspase-3, cleaved caspase-3, and actin antibodies. Proteolytic cleavage of PARP is indicated by the arrow (Right panel). (B) Apoptosis was analyzed as a sub-G1 fraction by FACS. The data is reported as the mean ± SD. (n = 3). * indicates P < 0.05 versus the miR-cont-transfected cells (Left panel). Immunoblots for PARP, procaspase-3, cleaved caspase-3, and actin. Proteolytic cleavage of PARP is indicated by the arrow (Right panel). (C) Apoptosis was analyzed as a sub-G1 fraction by FACS. The data is reported as the mean ± SD. (n = 3). * indicates P < 0.05 versus the miR-cont-transfected cells (Left panel). Immunoblots for PARP, procaspase-3, cleaved caspase-3, and actin. Proteolytic cleavage of PARP is indicated by the arrow (Right panel). **D.** Clonogenic assays performed as described in the Materials and Methods. **E.** The control pcDNA3.1 or c-FLIP_L_ expression vectors were transiently cotransfected into Caki cells with miR-708 and treated with indicated drugs for 24 h. The cells were harvested and analyzed by FACS (Upper panel) and Western blotting. Western blotting was performed using anti-c-FLIP and actin antibodies to confirm the transfection efficiency (Bottom panel). The data is reported as the mean ± SD (n = 3). * indicates P < 0.05 versus drug-treated vector cells transfected with pcDNA3.1.

Since c-FLIP_L_ inhibits the death receptor-mediated apoptotic pathway, we checked whether the reduction of c-FLIP_L_ expression by miR-708 could increase sensitivity to drugs that induce death receptor-mediated apoptosis in renal cancer cell lines. MiR-708-transfected cell lines were treated with TRAIL and cytotoxicity examined using fluorescence activated cell sorting (FACS). As shown in Fig. 4A, transfection with miR-708 resulted in a significant increase in the fraction of cells in the sub-G1 phase compared to miR-cont-transfected cells following treatment with TRAIL. We next investigated whether miR-708 could increase the sensitivity of renal cancer cells to apoptosis-inducing drugs such as thapsigargin (TG) and doxorubicin (Dox). Treatment of miR-708-transfected cells with TG and Dox resulted in a marked increase in the fraction of cells in the sub-G1 phase compared to cells expressing miR-cont as well as activation of caspase pathways (Fig. 4B and 4C). These results suggested that suppression of c-FLIP_L_ expression by miR-708 could increase the sensitivity of renal cancer cells to apoptosis-inducing agents. Clonogenic assays indicated that the numbers of surviving colonies treated with TRAIL, TG, and Dox in miR-708-transfected cells were much lower than those of miR-cont-transfected cells treated with TRAIL, TG, and Dox (Fig. 4D), demonstrating that transfection with miR-708 significantly reduced the reproductive potential of Caki cells treated with several apoptosis-inducing agents. MiR-708 plus TRAIL or Dox-induced apoptosis was prevented completely by pretreatment with the general and potent inhibitor of caspases, z-VAD-fmk, as determined by FACS analysis (Supplementary Fig. 2). These results indicated that the combined treatment of miR-708 plus TRAIL or miR-708 plus Dox result in activation of caspase-dependent apoptotic pathways.

Because miR-708 can inhibit c-FLIP_L_ expression by directly inhibiting the c-FLIP_L_ transcript, we next investigated whether reduction of c-FLIP_L_ expression could explain the increase in sensitivity of renal cancer cells to apoptosis-inducing agents. Therefore, miR-708 was ectopically expressed in Caki cells together with a construct containing the c-FLIP_L_ coding sequence but lacking the 3′-UTR of the c-FLIP_L_ mRNA or empty vector. The fraction of cells in the sub-G1 phase was lower in Caki/c-FLIP_L_-expressing cells compared to Caki/vector cells after treatment with TRAIL, TG, and Dox, indicating that the restoration of c-FLIP_L_ counteracted the effects of miR-708 on the sensitivity of renal cancer cells to apoptotic stimuli (Fig. 4E).

**Figure 5:**
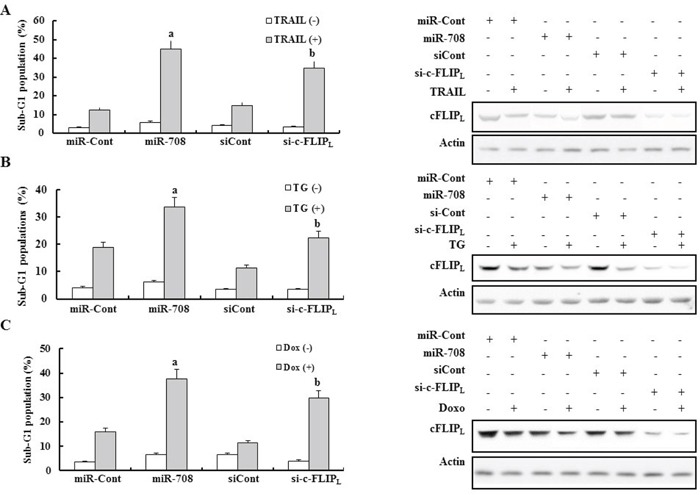
Downregulation of c-FLIP_L_ mediated by si-cFLIP_L_ enhanced sensitivity to various apoptotic stimuli Caki cells were transfected with miR-708 or si-c-FLIP_L_ with miR-Cont or si-Cont. Twenty-four hours after transfection, the cells were treated with TRAIL **A.** TG **B.** and Dox **C.** for 24 h. Apoptosis was analyzed as the sub-G1 fraction by FACS (Left panel). ^a^P < 0.05 compared to miR-Cont-transfected cells treated with the indicated drugs. ^b^P < 0.05 compared to si-Cont-transfected cells treated with the indicated drugs. Immunoblots for c-FLIP_L_ and actin (Right panel).

To determine whether the anti-cancer effects of miR-708 in renal cancer cell lines were due to c-FLIP_L_ inhibition or interaction with another gene, Caki cells were transiently transfected with a small interfering RNA (siRNA) specific to c-FLIP_L_ (si-c-FLIP_L_) or a scrambled siRNA negative control (si-Cont). The si-c-FLIP_L_ was able to knockdown expression of c-FLIP_L_ (Fig. 5A-5C, Left Panel). Depletion of c-FLIP_L_ by siRNA significantly increased the sensitivity of the cells to apoptosis-inducing drugs including TRAIL, TG, and Dox (Fig. 5A-5C, Right Panel). The anti-cancer effects of miR-708 were stronger than those of si-c-FLIP_L_ in TRAIL-, TG-, and Dox-treated cells (Fig. 5). These results could be explained by the fact that miR-708 can regulate multiple genes such as c-FLIP_L_, survivin, ZEB2, BMI1, and CD44, which are involved in separate tumor-suppressive pathways [26, 27]. We next conducted rescue experiments with survivin and c-FLIP_L_ to determine which protein was more important for enhancing the tumor-suppressive effect of miR-708 in renal cancer cells treated with anti-cancer drugs. Forced expression of c-FLIP_L_ and survivin demonstrated that the rescue effect of c-FLIPL is stronger than survivin in anticancer drug-treated cells (Supplementary Fig. 3).

To rule out the possibility of off-target effects of miR-708, we tested whether low concentrations of miR-708 achieved using another transfection system showed a similar trend. Ectopic expression of a low concentration of miR-708 inhibited c-FLIP_L_ expression (Supplementary Fig. 4A). In contrast, transfection with a low concentration of anti-miR-708 increased c-FLIP_L_ expression in Caki cells (Supplementary Fig. 4A). A low concentration of miR-708 reduced the luciferase activity of pmirGLO-c-FLIP #1 (Supplementary Fig. 4B). In addition, TRAIL, TG, and Dox treatment in low concentration miR-708-transfected cells resulted in a significant increase in the fraction of cells in the sub-G1 phase (Supplementary Fig. 4C).

### Restoration of miR-708 directly mediated the downregulation of c-FLIP_L_ expression *in vitro* and increased the sensitivity of renal cancer cells to various apoptotic stimuli

**Figure 6:**
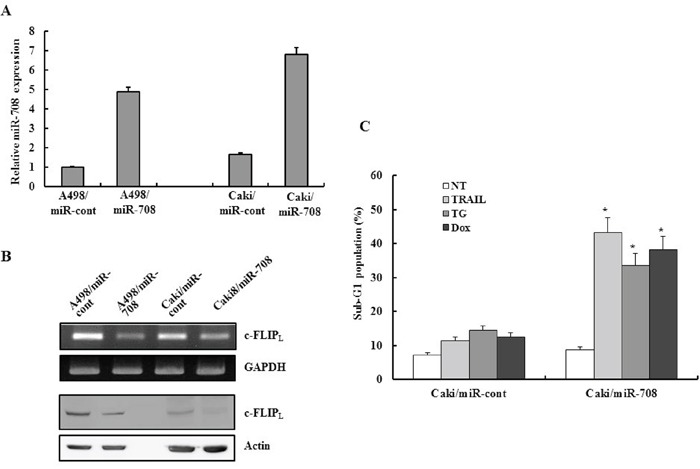
Restoration of miR-708 directly mediated downregulation of c-FLIP expression *in vitro* and increased the sensitivity of renal cancer cells to various apoptotic stimuli **A.** Quantitative RT-PCR analysis of the relative miR-708 expression levels in stably transfected A498 or Caki cells to confirm overexpression of miR-708 in the pooled cells. **B.** RT-PCR analysis and Western blotting to analyze the relative c-FLIP_L_ expression levels in stably transfected A498 or Caki cells. **C.** Caki/miR-cont and Caki/miR-708 cells were treated with the indicated drugs for 24 h. The cellular DNA content was measured after propidium iodide staining. The proportion of apoptotic cells is indicated. The data is reported as the mean ± SD (n = 3). *P < 0.05 versus Caki/miR-708 cells treated with the indicated drugs.

To determine whether miR-708 was directly related to downregulation of c-FLIP_L_, miR-708 overexpressing A498/miR-708 and Caki/miR-708 cells were established from A498 and Caki parent cells. While the A498/miR-708 and Caki/miR-708 cells exhibited an increase in miR-708 expression compared to cells containing empty vector, c-FLIP_L_ expression was suppressed in the A498/miR-708 and Caki/miR-708 cells compared to control cells (Fig. 6A and 6B). To examine the functional role of miR-708 in drug-mediated apoptosis in Caki cells, miR-708 overexpressing cell lines were treated with TRAIL, TG, and Dox for 24 h and the cytotoxicity examined. As shown in Fig. 6C, treatment of Caki/miR-708 cells with the drugs resulted in a marked increase in the fraction of cells in the sub-G1 phase compared to Caki/miR-cont cells. These results suggested that miR-708 restoration contributed to the phenotypic changes that endowed renal cancer cells with decreased expression of c-FLIP_L_ resulting in enhanced drug sensitivity.

### MiR-708 sensitizes xenograft tumors to a chemotherapeutic drug *in vivo*

**Figure 7:**
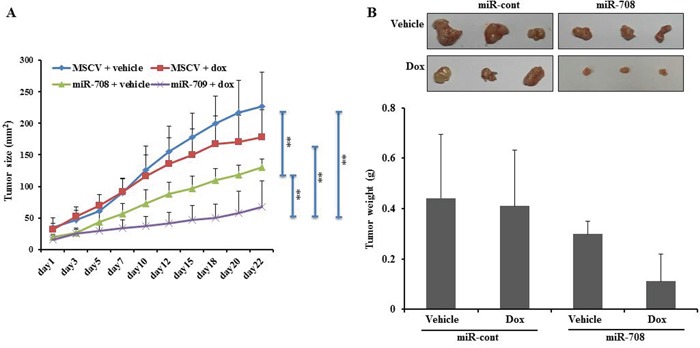
MiR-708 increases the sensitivity of tumor cells to Dox treatment *in vivo* **A.** Tumor size was compared between the four treatment groups and was calculated as πab/4 (a and b are the x and y diameters). MiR-708-overexpressing tumors showed greater sensitivity to Dox treatment than control mice. The results are expressed as the mean ± SD. Each point represents an average of seven mice. **B.** Tumor weights are plotted according to treatment groups. *P < 0.05 and ** P < 0.01.

To evaluate the effect of miR-708 on the sensitivity of renal cancer cells to a chemotherapeutic drug (Dox) *in vivo*, Caki cells stably overexpressing MSCV or miR-708 were injected into the right flanks of nude mice. When the tumors were palpable, the MSCV and miR-708 mice were subdivided into two groups and treated with either vehicle (water) or Dox (2 mg/kg). Consistent with previous reports, we found that ectopic expression of miR-708 inhibited tumor growth compared to miR-cont [26]. Importantly, within 3 weeks of treatment, miR-708 mice that received Dox had pronounced inhibition of tumor growth compared to MSCV mice that received the agent or miR-708 mice that received the vehicle control (Fig. 7). This indicates that miR-708 enhances the tumor-suppressive effect of Dox in a xenograft model of human RCC.

### The expression of MiR-708 and c-FLIP_L_ is inversely correlated in renal cancer tissues

**Figure 8:**
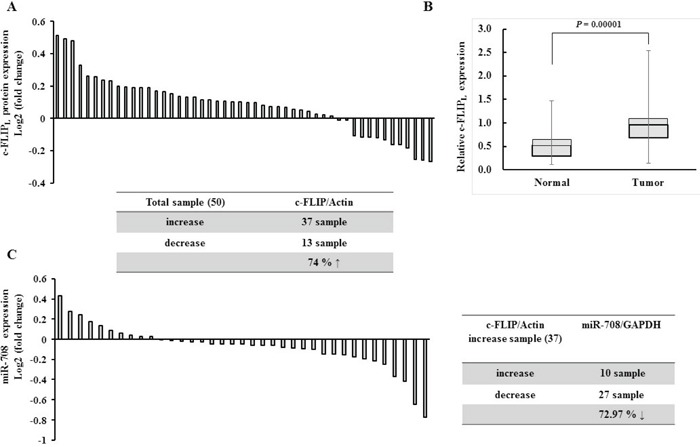
Expression of c-FLIP was inversely correlated with miR-708 expression in renal cancer tissues **A.** Relative c-FLIP_L_ protein expression levels in RCC clinical specimens and patient-matched normal tissues as assessed by Western blotting. **B.** Relative c-FLIP_L_ expression in normal versus tumor samples. Columns show c-FLIP_L_ expression in all normal and tumor tissue samples. The bars represent the SD. **C.** Relative miR-708 expression in c-FLIP_L_ overexpressing RCC clinical specimens and patient-matched normal tissues, as assessed by real-time PCR. The table summarizes relative c-FLIP_L_ protein and miR-708 expression in the RCC specimens.

As shown in Fig. 1, a significant inverse correlation was observed between c-FLIP_L_ and miR-708 expression in renal cancer and normal cell lines. Therefore, to determine whether there was an inverse relationship between c-FLIP_L_ and miR-708 expression in renal tissues, expression of c-FLIP_L_ was examined in 50 pairs of human renal tissue samples. The results indicated that c-FLIP_L_ was significantly overexpressed in renal cancer specimens compared to adjacent normal tissues (37 of 50 cases, 74%, Fig. 8A and Supplementary Fig. 5). The average c-FLIP_L_ expression in 50 renal cancer tissues specimens was higher than in adjacent normal tissues (Fig. 8B). As shown in Fig. 8C, miR-708 expression was significantly reduced in c-FLIP_L_ overexpressing RCC tissues compared to adjacent normal tissues (27 of 37 cases, 72.97%).

### MiR-708 specifically downregulates c-FLIP_L_ expression without affecting c-FLIP_s_ expression

**Figure 9:**
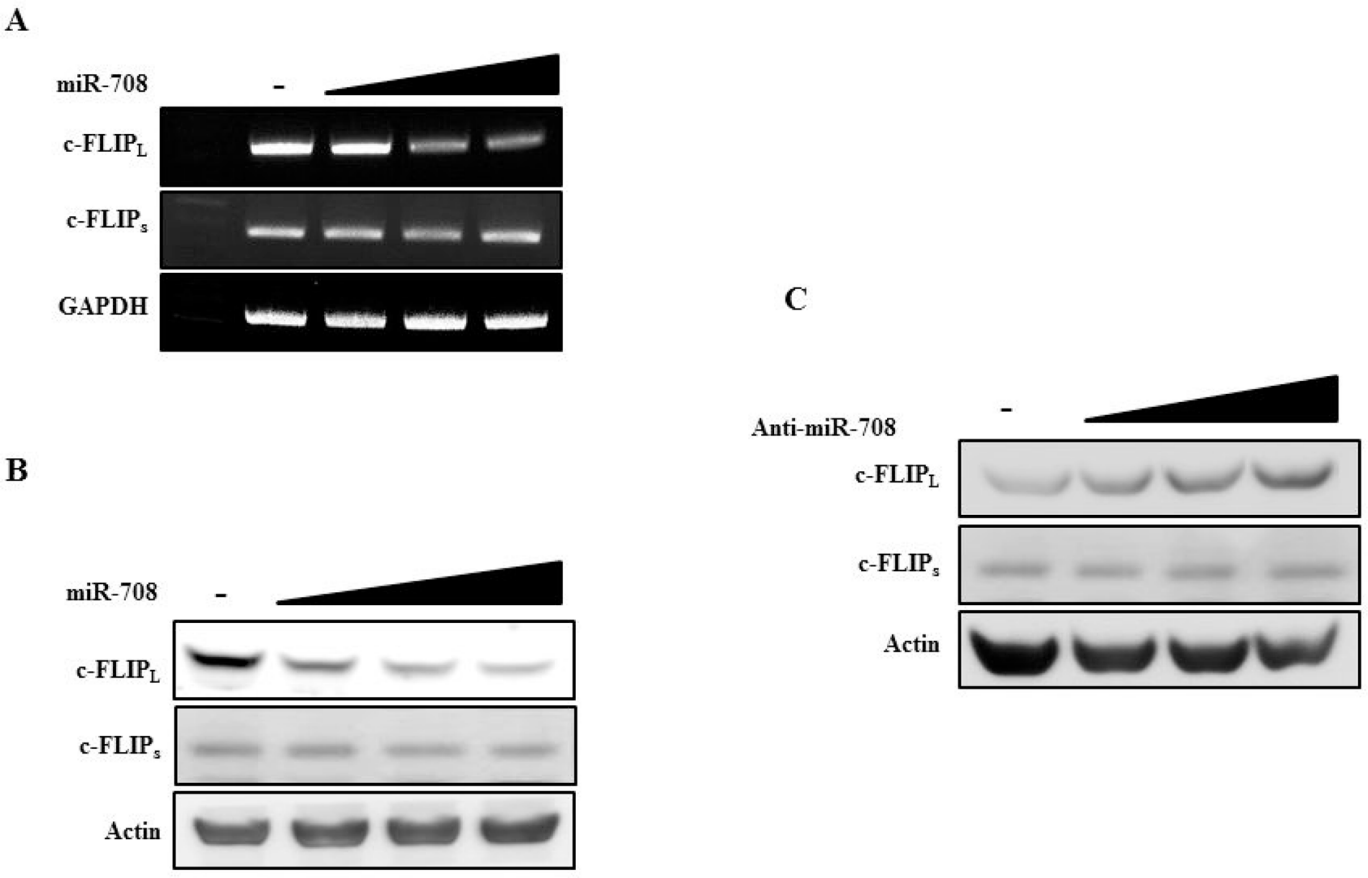
MiR-708 specifically downregulates c-FLIP_L_ expression without affecting c-FLIP_s_ expression **A.** RT-PCR analysis of c-FLIP_L_ and c-FLIP_s_ mRNA in Caki cells transfected as indicated. **B.** Immunoblots for c-FLIP_L_ and c-FLIP_s_ in Caki cells transfected as indicated. **C.** Immunoblots for c-FLIP_L_ and c-FLIP_s_ in Caki cells transfected as indicated. Actin was used as a loading control in all immunoblots.

The expression of c-FLIP_L_ and c-FLIP_s_ after transfection with miR-708 was analyzed to determine whether miR-708 specifically downregulated c-FLIP_L_ expression without affecting the levels of c-FLIP_s_. Transfection of Caki cells with miR-708 decreased c-FLIP_L_ expression. In contrast, no changes in the levels of c-FLIP_s_ mRNA and protein were detected in miR-708-transfected cells (Fig. 9A and 9B). As shown in Fig. 9C, transfection of Caki cells with anti-miR-708 increased c-FLIP_L_ protein expression but did not result in changes in c-FLIP_s_ expression. To investigate why miR-708 could repress c-FLIP_L_ expression but not c-FLIP_s_, we compared the miR-708 binding sequences in the 3′-UTRs of c-FLIP_L_ and c-FLIP_s_ using an alignment program. As shown in Supplementary Fig. 6, we did not find a miR-708 binding sequence in the 3′-UTR of c-FLIP_s_.

### MiR-708 downregulates c-FLIP_L_ expression in human renal cancer cells

**Figure 10:**
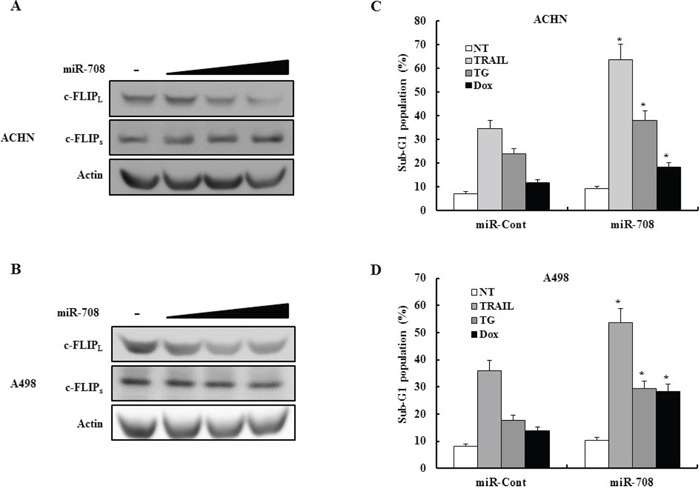
MiR-708 specifically downregulates c-FLIP_L_ expression without affecting c-FLIP_s_ expression in different renal cancer cells lines **A-B.** Immunoblots for c-FLIP_L_ and c-FLIP_s_ in ACHN cells (A) or A498 cells (B) transfected as indicated. Actin was used a loading control. **C-D.** ACHN (C) cells or A498 cells (D) were treated for 24 h with the indicated drugs and the cellular DNA content measured after propidium iodide staining. The proportion of apoptotic cells is indicated. The data is reported as the mean ± SD (n = 3). * indicates P < 0.05 versus miR-cont-transfected cells.

We investigated whether transfection of ACHN or A498 human renal cancer cells with miR-708 could inhibit expression of c-FLIP_L_ and c-FLIP_s_. As shown in Fig. 10A and 10B, transfection with miR-708 decreased c-FLIP_L_ expression without changing c-FLIP_s_ expression in both cell lines. We also examined whether transfection with miR-708 increased the sensitivity of renal cancer cells to several apoptotic stimuli. As shown in Fig. 10C and 10D, transfection with miR-708 significantly sensitized ACHN and A498 cells to TRAIL-, TG-, and Dox-induced apoptosis. Overall, these results indicated that direct suppression of c-FLIP_L_ expression is likely to be an efficient and attractive therapeutic strategy in RCC.

## DISCUSSION

Our study has demonstrated that c-FLIP_L_ is overexpressed in renal cancer cell lines and RCC tissues compared to normal cells and tissues. In addition, we showed that ectopic expression of miR-708 decreased c-FLIP_L_ mRNA and protein levels in renal cancer cells, which was caused by direct binding of miR-708 to the miR-708 binding site of the c-FLIP_L_ 3′-UTR. Transfection with miR-708 induced cell death in a time-dependent manner and enhanced the sensitivity of Caki cells to apoptosis-inducing drugs such as TRAIL, TG, and Dox. Consistent with our *in vitro* results, miR-708 significantly enhanced the tumor-suppressive activity of Dox in nude mice. Moreover, restoration of c-FLIP_L_ counteracted the effects of miR-708 on the sensitivity of Caki cells to apoptotic stimuli. In RCC, expression of c-FLIP_L_ and miR-708 was found to be inversely correlated both *in vitro* and *in vivo* (i.e., c-FLIP_L_ was upregulated while miR-708 was rarely expressed).

Overexpression of c-FLIP has been observed in several types of malignancies including colorectal carcinoma, hepatocellular carcinoma, pancreatic carcinoma, and prostate carcinoma, and might be associated with cancer progression given the ability of c-FLIP to inhibit apoptosis [16, 28–31]. In the present study, c-FLIP_L_ expression in RCC tissues was higher than in adjacent normal tissues. In this study, we also searched for novel miRNA that targeted c-FLIP_L_ in renal cancer cells using online databases such as Targetscan. The results indicated that the c-FLIP_L_ mRNA contained miR-708 binding sites. As expected, miR-708 could bind to the c-FLIP_L_ 3′-UTR in renal cancer cells and decrease its expression, raising the possibility that miR-708 might act as a tumor suppressor. The function of miR-708 in cancer is controversial. It acts as an oncogene by contributing to tumor growth and disease progression through downregulation of TMEM88 in lung cancer [32]. It also has been shown to promote bladder cancer growth and inhibit apoptosis by targeting caspase-2 [33]. In contrast, miR-708 has been shown to induce apoptosis and suppresses tumorigenicity via regulation of survivin in RCC [26]. In addition, miR-708 was consistently downregulated in prostate cancer cell lines, which resulted in prostate cancer development and progression through regulation of CD44 and Akt2 expression [27]. These studies suggest that miR-708 may act as a tumor suppressor in cancer cells. Real-time RT-PCR was used to examine miR-708 expression in 50 different RCC tissues. Among these tissues, 27 of the 50 cases (54%) showed lower miR-708 levels relative to matched normal tissues. This is in accordance with the observations of Saini et al. [26], who reported that miR-708 was downregulated in RCC tissue samples (20 of 38, 53%). Moreover, the miR-708 levels in this study were reduced in 27 out of 37 tissue samples (72.97%) that overexpressed c-FLIP_L_. Expression of c-FLIP_L_ was inversely correlated with miR-708 expression in renal cancer cell lines and RCC tissues, further supporting the idea that miR-708 directly downregulates c-FLIP_L_ expression in RCC. These results support the concept that miR-708 acts as a tumor suppressor by repressing c-FLIP_L_ expression in RCC.

Because c-FLIP inhibits death receptor- or anti-cancer drug-mediated apoptosis as well as activates several cytoprotective signaling pathways involved in regulating survival, proliferation, and carcinogenesis, targeting c-FLIP may be an attractive strategy for anti-cancer therapy. Many studies have clearly indicated that selective inhibitors of c-FLIP in combination with a death ligand such as TRAIL or a conventional chemotherapy such as 5-fluorouracil and oxaliplatin can be an effective antitumor therapy [11–34]. In this study, we also examined whether suppression of c-FLIP_L_ by miR-708 could increase the sensitivity of renal cancer cells to death ligands or conventional chemotherapy. The results showed that miR-708 increased the sensitivity to TRAIL, suggesting that miR-708 is a regulator of TRAIL-mediated apoptotic pathways in renal cancer cells. In addition, miR-708 sensitized renal cancer cells to Dox and TG by inhibiting expression of c-FLIP_L,_ and restoration of c-FLIP_L_ expression attenuated Dox- and TG-mediated apoptosis in miR-708-transfected cells. These results suggested that downregulation of c-FLIP_L_ by miR-708 was involved in anti-cancer drug-mediated apoptosis in renal cancer cells. In a previous report, miR-708 was shown to induce apoptosis via repression of survivin expression in renal cancer cells [26]. Therefore, we evaluated which protein had the more important role in enhancing the tumor-suppressive effect of miR-708 in anti-cancer drug-treated renal cancer cells. We found that c-FLIP_L_ contributed to miR-708 induced-drugs sensitization to a greater extent than survivin.

Owing to the high structural homology between c-FLIP and procaspase-8, small molecules that target c-FLIP are also capable of blocking the recruitment of caspase-8 to the DISC. Therefore, it is important to develop compounds that inhibit or downregulate c-FLIP mRNA expression or cause degradation of c-FLIP protein without inhibiting caspases-8. Two isoforms of c-FLIP (the long and short forms) exist as a result of alternative splicing. Expression of the c-FLIP isoforms is regulated at the transcriptional, translational, and post-translational levels [35–37]. In addition, expression of the c-FLIP isoforms would need to be specifically regulated for cancer therapy because c-FLIP_L_ and c-FLIP_s_ play different roles in cell death pathways including apoptosis, necroptosis, and autophagy [21–25]. For instance, c-FLIP isoforms exert opposite effects on ripoptosome formation. Ripoptosome formation is inhibited by c-FLIP_L_. As a consequence, c-FLIP_L_ blocks apoptosis and maintains cell survival. In contrast, c-FLIP_s_ promotes ripoptosome assembly, undergoing necroptotic cell death. Autophagy is also attenuated by c-FLIP_L_, which competed with Atg3 binding to LC3 thereby decreasing LC3 processing and inhibiting autophagosome formation. Therefore, a deeper understanding of the how the expression of c-FLIP isoforms is regulated and the functions of the different isoforms is necessary to enable the rational design of selective c-FLIP inhibitors. Although many drugs have been shown to sensitize tumor cells to cell death by inhibiting expression of c-FLIP, small molecules that directly target c-FLIP mRNA have rarely been reported due to a lack of information on post-transcriptional regulation of c-FLIP. Recent data have indicated that the inhibition of c-FLIP expression by miR-512-3p contributes to taxol-induced apoptosis in hepatocellular carcinoma cells [19]. Based on these concepts, we screened for novel miRNAs that specifically suppressed c-FLIP_L_ expression without affecting c-FLIP_s_ expression in renal cancer cells. Using Western blotting and RT-PCR assays, we demonstrated that miR-708 failed to reduce c-FLIP_s_ mRNA and protein levels, suggesting that miR-708 specifically suppresses c-FLIP_L_ expression with little effect on c-FLIP_s_ expression.

In conclusion, c-FLIP_L_ expression was upregulated while miR-708 expression was downregulated in RCC tissues compared to normal tissues. Additionally, miR-708 functioned as a pro-apoptotic miRNA via specific downregulation of c-FLIP_L_ expression but did not have any effect on the expression of c-FLIP_s_, which can also increase the drug sensitivity of renal cancer cells. In xenograft mice bearing human RCCs, miR-708 suppressed tumor growth and enhanced the tumor regression activity of Dox. These results improve our understanding of the molecular mechanisms underlying renal cell tumorigenesis, the regulation of the c-FLIP isoform expression, and the roles of the different isoforms in cell death pathways. Direct inhibition of c-FLIP_L_ expression could be a potent strategy for the treatment of RCC.

## MATERIALS AND METHODS

### Sample population

Fifty pairs of primary renal cancer tissues and adjacent non-cancerous tissues were obtained from the Department of Pathology and the National Biobank of Korea, Pusan National University Hospital (PUNH). All samples were reviewed for diagnostic accuracy by a pathologist. This study was approved by the Institutional Review Board at PUNH.

### Cells and materials

Caki cells were obtained from the American Type Culture Collection (ATCC: Rockville, MD, USA). The culture medium used for all experiments consisted of Dulbecco's modified Eagle Medium supplemented with 10% fetal bovine serum, 20 mM HEPES buffer, and 100 μg/mL gentamicin. The anti-Bcl-2, -procaspase-3, -PARP, and -actin antibodies were purchased from Santa Cruz Biotechnology Inc. (Santa Cruz, CA, USA). The anti-c-FLIP antibody was purchased from ALEXIS Corporation (San Diego, CA, USA). Soluble recombinant TRAIL was purchased from R&D Systems (Minneapolis, MN, USA). TG and Dox were obtained from Sigma Chemical Co (Saint Louis, MO). Anti-DR5 was purchased from KOMA Biotech Inc. (Seoul, Korea).

### Western blotting

The cellular lysates were prepared by suspending 5×10^5^ cells in 100 μL of lysis buffer (137 mM NaCl, 15 mM EGTA, 0.1 mM sodium orthovanadate, 15 mM MgCl_2_, 0.1% Triton X-100, 25 mM MOPS, 100 μM phenylmethlsulfonyl fluoride, and 20 μM leupeptin, pH 7.2). The cells were disrupted by sonication and extracted at 4°C for 30 min. The total protein in the lysates was quantified using the BCA Protein Assay Kit (Pierce, Rockford, IL, USA). The proteins were electrotransferred to Immobilon-P membranes (Millipore, Bedford, MA, USA). Detection of specific proteins in the lysates was performed using an ECL Western Blotting Detection Kit (Millipore) according to the manufacturer's instructions.

### Cell counts and flow cytometry analysis

Cell counts were performed using a hemocytometer. Approximately 1 × 10^6^ cells were suspended in 100 μL of PBS and 200 μL of 95% ethanol was added while vortexing. The cells were incubated at 4°C for 1 h, washed with PBS, and then resuspended in 250 μL of 1.12% sodium citrate buffer (pH 8.4) together with 12.5 μg of RNase. The cells were then incubated for an additional 30 min at 37°C. Cellular DNA was stained with 250 μL of propidium iodide (50 μg/mL; PI; Sigma, St. Louis, MO, USA) for 30 min at room temperature. The cells were then analyzed by FACS on a FACScanto flow cytometer to determine the relative DNA content based on fluorescence.

### RNA isolation and RT-PCR

Total cellular RNA was extracted from the cells using an Easy-Blue Total RNA Extraction Kit (Intron, SungNam, Korea). The cDNA was synthesized from 5 μg of total RNA using M-MLV reverse transcriptase (Promega, Madison, WI, USA). The cDNA for c-FLIP_L_ and actin were amplified by PCR with specific primers. The sequences of the sense and antisense primers for c-FLIP_L_ were 5′-CGGACTATAGAGTGCTGATGG-3′ and 5-GATTATCAGGCAGATTCCTAG-3′ (c-FLIP_L_), respectively. The PCR products were analyzed by agarose gel electrophoresis and visualized with ethidium bromide staining.

### Real-time quantitative RT-PCR

Real-time quantitative RT-PCR was performed to detect the levels of miR-708 expression in renal cancer tissues and matched adjacent normal tissues. Small RNAs were extracted using the miRNEasy RNA Isolation Kit (Qiagen, Germantown, MD, USA) according to the manufacturer's instructions. The RT reactions were performed using 20 ng of small RNA extracted from the tissues using a TaqMan^®^ MicroRNA Reverse Transcription Kit and TaqMan^®^ MicroRNA assays specific to the mature form of hsa-miR-708. Real-time PCR reactions were performed using the TaqMan Universal PCR Master Mix No AmpErase UNG (Applied Biosystems, Foster City, CA, USA). The amplification reactions were run on an ABI PRISM 7000 Sequence Detection System (Applied Biosystems).

### Luciferase reporter assays

For the basic 3′-UTR luciferase reporter assay, Caki cells were transfected with the c-FLIP_L_ 3′-UTR-pmirGLO Dual-Luciferase reporter plasmid (Promega), miR-cont, miR-708, miR-708 mimics, or anti-miR-708 using Lipofectamine 2000. Luciferase activity assays were then performed and normalized to Renilla luciferase activity. The experiments were repeated three times.

### Stable genetic modulation of miR-708 in renal cancer cell lines

A PCR fragment of approximately 600 base pairs encompassing the miR-708 precursor sequence was cloned into a Murine Stem Cell Virus (MSCV)-puro retrovirus vector. Generation of retroviral particles, transduction, and selection with puromycin (0.75 μg/mL) were performed as described previously. The cloning primers were as follows: 5′-AGATCTTTTCCAGCAATGCAAAAGTG-3′ (MSCV-miR-708-forward) and 5′-GAATTCTGGCCCAGAGGAGATATGAC-3′ (MSCV-miR-708-reverse).

### Xenograft studies

Twenty eight nude mice (BALB/c-nu; 5 weeks old; Central Lab. Animal Inc.) were divided randomly into two cohorts and inoculated in the right flank with 2 × 10^6^ Caki cells that stably expressed MSCV or MSCV-miR-708. Once palpable tumors formed, each cohort was subdivided into two groups and treated with the vehicle or doxorubicin (2 mg/kg). Treatments were administered twice a week intraperitoneally and tumors were measured every 2–3 days using a digital caliper. Tumor size was calculated as described previously [38]. After 3 weeks of treatment, the mice were sacrificed humanely and the tumors were weighed. The animal protocol used in this study was reviewed and approved by the Pusan National University-Institutional Animal Care and Use Committee for ethical procedures and scientific care.

### Statistical analysis

Three or more independent experiments were performed and comparisons between the experimental and control groups were conducted. Statistical analyses were performed using the paired Student's t-test or ANOVA. A P value < 0.05 was considered significant.

## SUPPLEMENTARY DATA, FIGURES


