# Supplementary material for: Inhibition of c-FLIPL expression by miRNA-708 increases the sensitivity of renal cancer cells to anti-cancer drugs
Source: Oncotarget. 2016 Feb 3;7(22):31832–46. doi: 10.18632/oncotarget.7149 (PMC5077980; doi:10.18632/oncotarget.7149)
Supplement: Supplementary file 1 [file oncotarget-07-31832-s001.pdf]

# Inhibition of c-FLIP$_L$ expression by miRNA-708 increases the sensitivity of renal cancer cells to anti-cancer drugs

## SUPPLEMENTARY DATA

### Clonogenic assay

Cells were plated in a 6-well plate and transfected with miR-708 and miR-cont. After 72 h of transfection, cells were subcultured in a new 6-well plate (500–1,000/well) for 14 days. Colonies were stained with 0.5% Methylene blue and counted. All experiments were performed in triplicate wells.

## SUPPLEMENTARY FIGURES

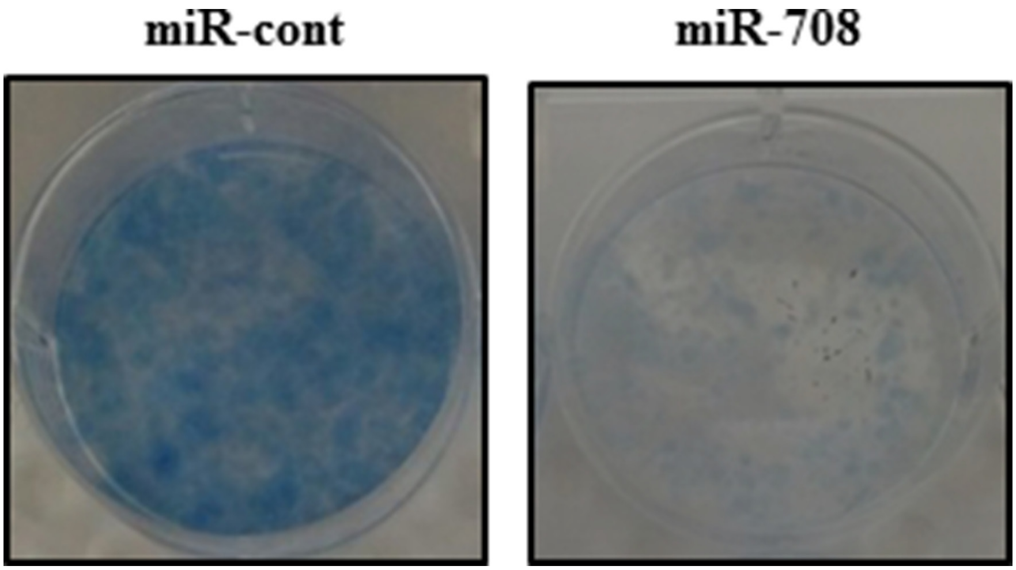


**Supplementary Figure S1: Effect of miR-708 on reproductive potential of the Caki cells.** After Caki cells were transiently transfected with miR-708 or miR-cont for 72 h, clologenic assay was performed as previously described as mentioned above.

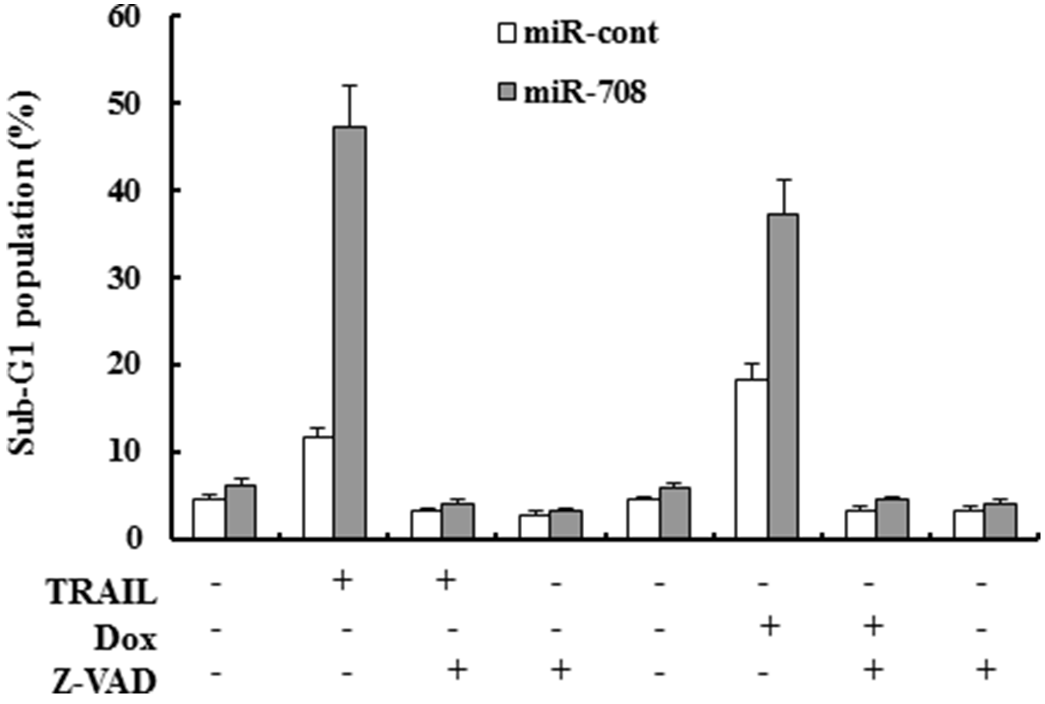


**Supplementary Figure S2: Effect of z-VAD-fmk on apoptosis induced by miR-708 plus TRAIL or Dox.** The Caki cells were incubated with 50 µM z-VAD-fmk or the solvent for 1 h before treatment with miR-708 and/or TRAIL and Dox for 24 h. DNA contents of the treated cells were evaluated after propidium iodide staining and apoptosis was measured as a sub-G1 fraction by FACS. The data is reported as the mean values obtained from three independent experiments and bars represent standard deviation.

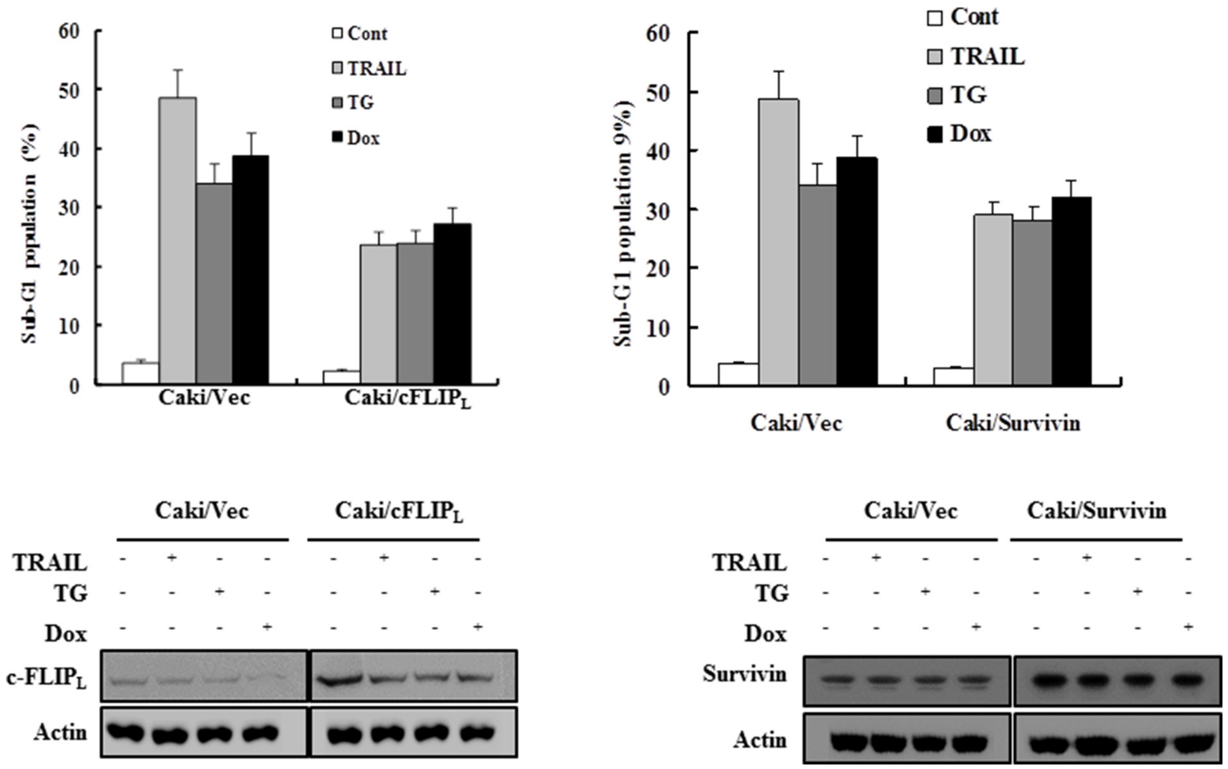


**Supplementary Figure S3: The forced expression of c-FLIP$_L$ and survivin demonstrated that c-FLIP$_L$ appeared to contribute to miR-708 induced-drugs sensitization more than survivin.** The control pcDNA3.1 or c-FLIP$_L$ expression vectors were transiently cotransfected with miR-708 and treated with indicated drugs in Cake cells for 24 h. The cells were harvested and analyzed by FACS (Upper panel) and Western blotting. Western blotting was performed using anti-c-FLIP and actin antibodies to confirm the transfection efficiency (Bottom panel). The data is reported as the mean ±SD (n = 3). * indicates P <0.05 versus drugs-treated vector cells that are transfected with pcDNA3.1.

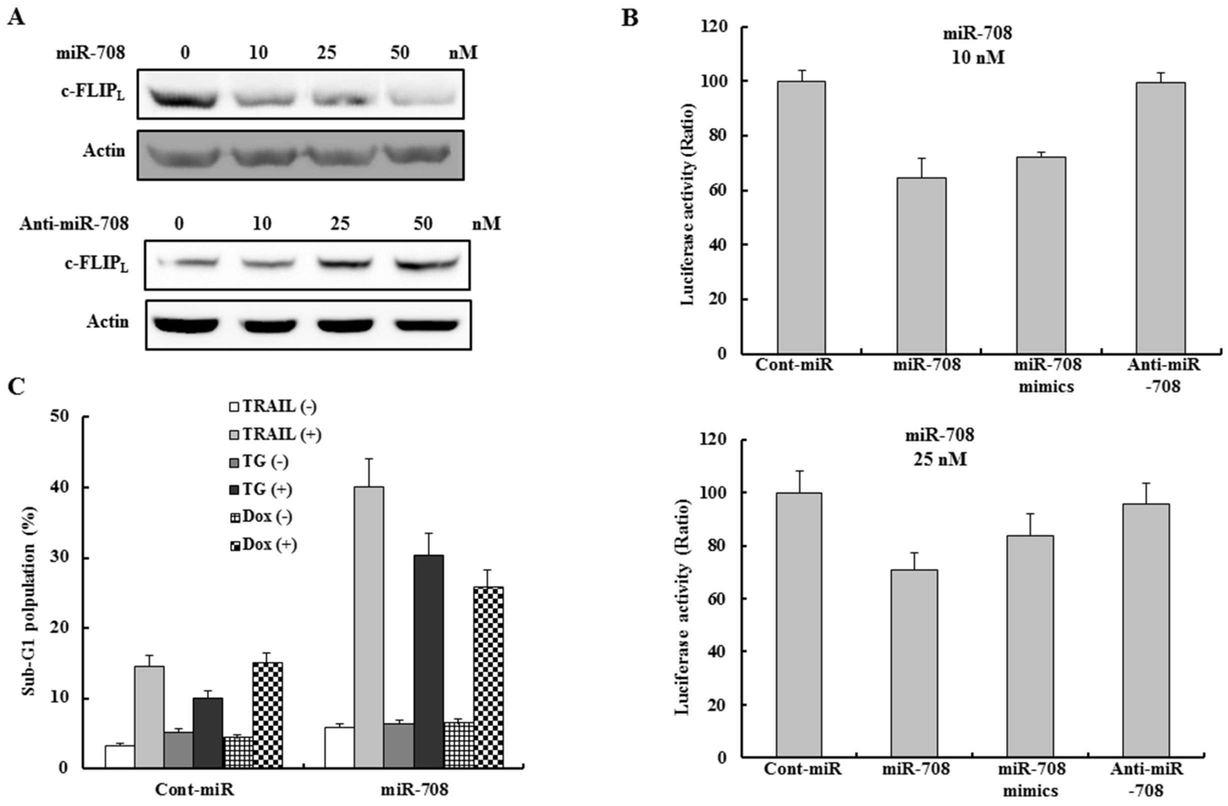


**Supplementary Figure S4: Transfection with the low concentration of miR-708 using Lipofectamine® RNAiMAX Reagent showed the same trend as it showed on results.** To improve the transfection efficiency, we used Lipofectamine® RNAiMAX Reagent (Invitrogen). **A.** Immunoblots for the c-FLIPL protein in Caki cells transfected as indicated. **B.** Luciferase activity assay with the respective wild-type luciferase constructs containing miR-708 target sequences (site #1) transfected with miR-cont or miR-708 (10 nM or 25 nM). **C.** Caki cells transfected with miR-cont or miR-708 (25 nM) were treated with TRAIL, TG, or Dox for 24 h and their DNA content was measured after propidium iodide staining.

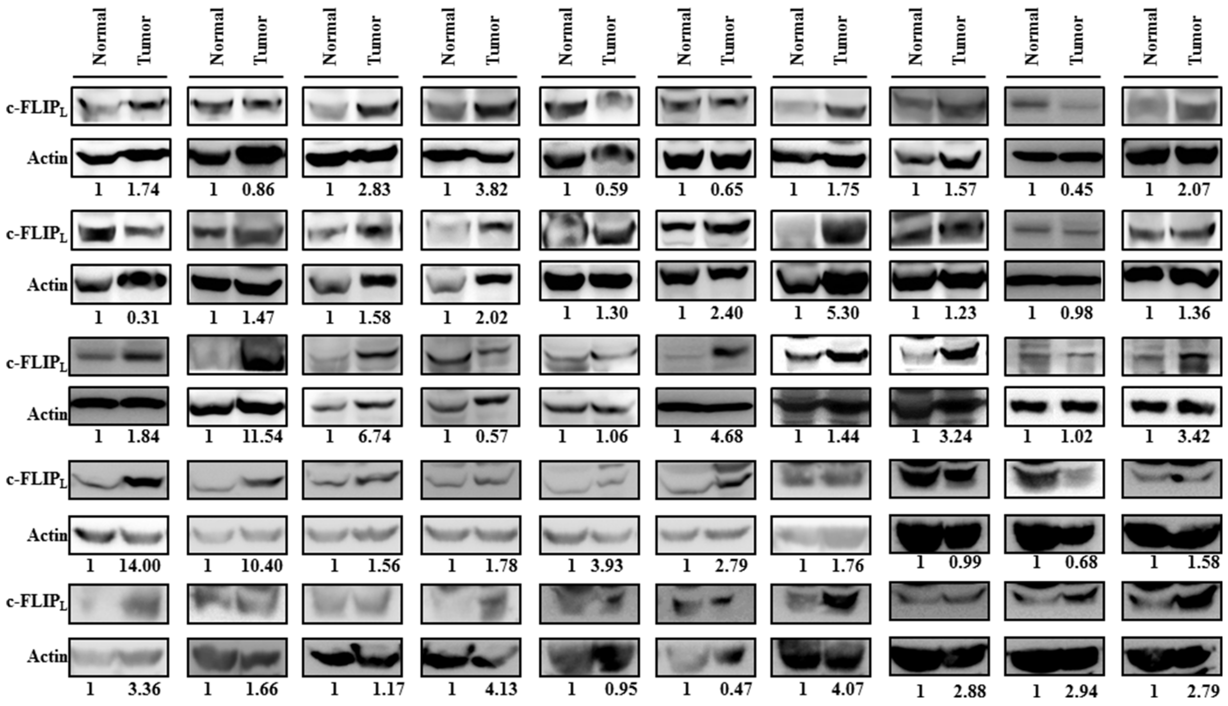


**Supplementary Figure S5: c-FLIP$_L$ protein expressions in the paired human renal cancer and normal tissue.**

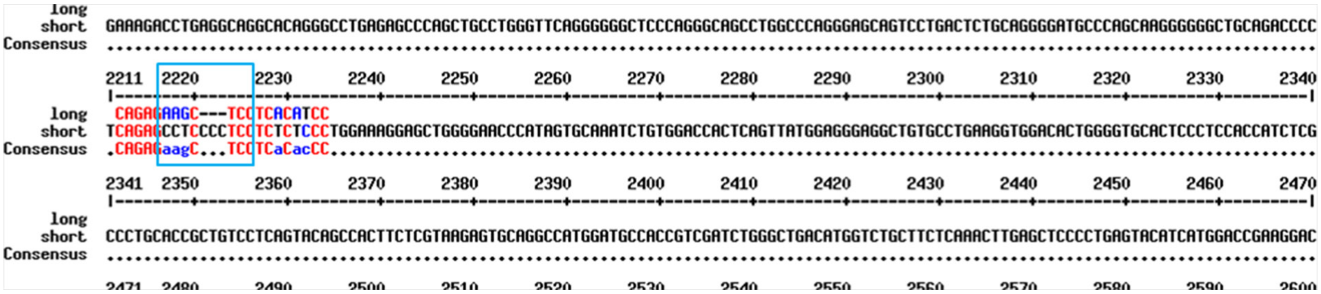

**Supplementary Figure S6: Alignment of miR-708 binding sequences of c-FLIP$_L$ 3'-UTR and c-FLIPs 3'-UTR.** Blue boxes indicate regions of miR-708 binding sequence in c-FLIP$_L$ 3'-UTR. Long; miR-708 binding sequence in c-FLIP$_L$ 3'-UTR, Short: c-FLIP$_s$ 3'-UTR.
